# Smad Mediated Regulation of Inhibitor of DNA Binding 2 and Its Role in Phenotypic Maintenance of Human Renal Proximal Tubule Epithelial Cells

**DOI:** 10.1371/journal.pone.0051842

**Published:** 2013-01-08

**Authors:** Mangalakumar Veerasamy, Mysore Phanish, Mark E. C. Dockrell

**Affiliations:** South West Thames Institute for Renal Research, St.Helier University Hospital NHS Trust, Carshalton, United Kingdom; Fondazione IRCCS Ospedale Maggiore Policlinico & Fondazione D'Amico per la Ricerca sulle Malattie Renali, Italy

## Abstract

The basic-Helix-Loop-Helix family (bHLH) of transcriptional factors plays a major role in regulating cellular proliferation, differentiation and phenotype maintenance. The downregulation of one of the members of bHLH family protein, inhibitor of DNA binding 2 (Id2) has been shown to induce de-differentiation of epithelial cells. Opposing regulators of epithelial/mesenchymal phenotype in renal proximal tubule epithelial cells (PTEC), TGFβ1 and BMP7 also have counter-regulatory effects in models of renal fibrosis. We investigated the regulation of Id2 by these growth factors in human PTECs and its implication in the expression of markers of epithelial versus myofibroblastic phenotype. Cellular Id2 levels were reduced by TGFβ1 treatment; this was prevented by co-incubation with BMP7. BMP7 alone increased cellular levels of Id2. TGFβ1 and BMP7 regulated Id2 through Smad2/3 and Smad1/5 dependent mechanisms respectively. TGFβ1 mediated Id2 suppression was essential for α-SMA induction in PTECs. Although Id2 over-expression prevented α-SMA induction, it did not prevent E-cadherin loss under the influence of TGFβ1. This suggests that the loss of gate keeper function of E-cadherin alone may not necessarily result in complete EMT and further transcriptional re-programming is essential to attain mesenchymal phenotype. Although BMP7 abolished TGFβ1 mediated α-SMA expression by restoring Id2 levels, the loss of Id2 was not sufficient to induce α-SMA expression even in the context of reduced E-cadherin expression. Hence, a reduction in Id2 is critical for TGFβ1-induced α-SMA expression in this model of human PTECs but is not sufficient in it self to induce α-SMA even in the context of reduced E-cadherin.

## Introduction

The inhibitor of DNA binding 2 (Id2) protein belongs to the basic-Helix-Loop-Helix (bHLH) family of transcriptional regulators which are involved in cell cycle entry and proliferation [1], survival [2], differentiation and lineage commitment [3], [4]. The members of the bHLH proteins form homo or heterodimers with other bHLH proteins through their conserved HLH domain and these bHLH dimers regulate the gene expression by binding through their basic region with the E-box sequence (CANNTG) in the promoter region of the target gene [5]. Though Id family of proteins posses HLH domain, they lack the DNA binding basic region hence when they form a dimer with the other bHLH proteins they inhibit the E-box binding activity of the other bHLH proteins [5], [6].

Phenotypic transition of differentiated epithelial cells has been studied extensively in the context of pathological tissue fibrosis. Although it has been disputed [7], the transition of epithelial phenotype to a mesenchymal phenotype (EMT) is considered as one of the sources of matrix secreting fibroblasts in fibrosis involving vital organs like kidney, liver and lung [8]–[10]. With regard to renal fibrosis, one report suggested that proximal tubule epithelial cells (PTECs) contributed to 36% of the total fibroblasts pool through EMT [11]. Although a growing body of evidence from both *in vitro* and animal studies confirm the occurrence of EMT in renal epithelial cells [12]–[14], the reports from human samples are sparse [8], [15].

In addition to EMT, mesenchymal transition of endothelial cells [16] and bone marrow derived mesenchymal cells have also been shown to contribute to renal fibrosis. Recently, the Duffield group [7], [17] demonstrated pericytes to be the major source of renal interstitial fibroblasts and these results question the validity of EMT as a precursor of interstitial fibroblasts. In fibrotic kidneys the matrix is composed of a number of constituents that include collagen I, collagen III, fibronectin etc. Whether all these matrix proteins are secreted by one fibroblast population or fibroblasts arising from different sources secrete different matrix proteins has not been addressed. One could hypothesise that the origin of fibroblasts is variable depending on the model studied and they could secrete different matrix constituents.

The phenotypic transition of epithelial cells involves coordinated involvement of multiple signalling pathways [18]. The loss of E-cadherin (a marker of epithelial phenotype) and *de novo* acquisition of α-SMA are considered as important features of phenotype transition process of epithelial cells [13]. Both E-cadherin and α-SMA genes have E-box elements in their promoter region and E-box binding bHLH proteins like E12 and E47 have been implicated in the regulation of the expression of both of them [19], [20]. Id2 has been shown to prevent the downregulation of E-cadherin in epithelial cell models (NMuMG, HaCaT keratinocytes) [21]. In these cell models Id2 was found to suppress α-SMA expression after TGFβ1 stimulation- a well characterised pro-fibrotic growth factor and inducer of EMT [22].

TGFβ1 regulates the expression of markers of EMT through activating predominantly Smad2/3 signalling in human renal PTEC model (HKC 8) [23]. In these cells TGFβ1 induced Id1 expression, another member of Id family through Smad2/3 signalling and this resulted in E-cadherin loss [14]. However Id1 upregulation was not involved in TGFβ1 induction of α-SMA. In contrast to TGFβ1, BMP 7 the other member of TGF family has been shown to have anti-fibrotic effect in both *in vivo* and *in vitro* models of renal fibrosis [24]–[26]. One of the mechanisms by which BMP 7 exerts its anti-fibrotic effect is by inhibiting TGFβ1 mediated E-cadherin loss and subsequent development of EMT at least in murine models [24]. The latter result has not been consistently replicated in human models [27].

We have earlier reported in HKC 8 cells that BMP 7 inhibited TGFβ1 mediated α-SMA expression as well as fibronectin secretion through activating Smad1/5 signalling [28], [29]. Though published reports support the role of Id2 in the regulation of E-cadherin and α-SMA, it has not been studied in the context of TGFβ1 and BMP 7 in human renal PTECs. Since these growth factors play a pivotal role in the regulation of renal fibrosis by regulating PTEC phenotype, we investigated the regulation of Id2 by these two growth factors in relation to the expression of key markers of EMT. In particular we investigated whether counter-regulation of Id2 by BMP 7 may account for its anti-fibrotic effect by inhibiting TGFβ1 mediated expression of α-SMA and the myofibroblastic transition of PTECs.

## Methods

### Cell Culture

All the experiments were performed in SV40 transformed human renal PTECs (HKC 8) provided by Dr. L Racusen of John Hopkins University (Baltimore, Md, USA) [30]. The cell culture method has been described earlier [28]. In brief, HKC 8 cells were grown on uncoated plastic ware in DMEM/F12 medium (Dulbecco's modified Eagle's medium 1∶1 v/v) enriched with supplements (tri-iodothyronine (20 ng/ml), hydrocortisone (18 ng/ml), insulin (5 µg/ml), sodium selenite (5 ng/ml), transferrin (5 µg/ml) and 5% (v/v) heat inactivated fetal bovine calf serum (FCS)) at 37°C in an incubator with 5%CO_2_ and 95% humidified air. HKC 8 cells were sub-cultured by trypsin treatment (0.5% porcine trypsin-5.3 mM EDTA•4Na) for 3 min at 37°C and the viable cells were counted after trypan blue staining. The cells were seeded at a density of 10^4^ cells/cm^2^ in 35 mm cell culture dishes for all the experiments unless stated otherwise. In all the instances the cells were serum deprived for 24 h prior to growth factor stimulation, then treated with medium (0.1% bovine serum albumin (BSA)), TGFβ1 (5 ng/ml), recombinant human BMP 7 (200 ng/ml) or a combination of both TGF β1(5 ng/ml) and BMP 7 (200 ng/ml). The concentration of TGFβ1 used was based on our earlier work, that this was the minimum concentration required to induce full EMT [23], [31], [32]. Similarly, we found that BMP 7 induced phosphorylation of Smad1/5/8 reached its peak at the concentration of 200 ng/ml and beyond this there was no incremental activation seen. Hence we used this concentration of BMP 7 in all experiments to study the Smad1/5/8 dependent events. The expression of markers of EMT was studied 48 h after growth factor stimulation based on our previous work that this was the minimum time required to induce the major phenotypic features of EMT [23].

### Materials

Tissue culture medium, cell culture supplements and the immunoblotting systems were obtained from Invitrogen (Gibco, Paisley, UK) and Sigma-Aldrich (Sigma-Aldrich, Poole, UK). Small interfering RNAs (siRNA) and transfecting reagent were obtained from Invitrogen (Invitrogen, Paisley, UK). Anti-E-cadherin (clone 36) antibody was purchased from BD Transduction Laboratories, anti-Smad1 (#9512), anti-Smad2 (#3122), anti-Smad3 (#9513), anti-Smad5 (#9517) and goat anti-rabbit horseradish peroxide (HRP) conjugated (#7074) antibodies were from Cell Signaling Technology (New England Biolabs (UK) Ltd, Hitchin, UK). Anti-α-tubulin (T6199), anti-α-smooth muscle actin (A2547) and anti-mouse HRP conjugated antibodies (A9044) were from Sigma-Aldrich (Sigma-Aldrich, Poole, Dorset, UK). Anti-Id2 antibody (sc-489) was purchased from Santa Cruz Biotechnology (Autogen Bioclear UK, Calne, UK). Human recombinant TGFβ1 (T7039) was purchased from Sigma-Aldrich (Sigma-Aldrich, Poole, Dorset, UK) and human recombinant BMP 7 (354-BP) was purchased from R&D systems (R&D systems Europe Ltd, Abingdon, UK).

### Immunoblotting

After washing with cold phosphate buffered saline (PBS) the cells were lysed with freshly made lysis buffer (20 mM Tris HCL, 150 mM NaCl, 1% Triton X-100, 0.5% sodium deoxycholate, 0.1% sodium dodecyl sulfate, 2 mM EDTA, 1.0 mM sodium orthovanadate, 50 mM sodium fluoride, 200 µM phenylmethanesulfonyl fluoride and 40 µl/ml of protease inhibitor cocktail (Sigma-Aldrich)). After complete cell lysis, the lysate was centrifuged at 4°C for 10 min at 10000 *g* to separate the supernatant containing cellular proteins. The protein content of the lysate was measured by Bicinchoninic acid (BCA) protein assay (Pierce, Perbio Science UK Ltd, Cramlington, UK). The cellular proteins were subjected to SDS/PAGE in 12% w/v Bis-Tris containing polyacrylamide gels under reducing conditions according to the manufacturers' instructions and the separated proteins were then transferred to a PVDF membrane. The membrane was blocked with 5% w/v fat free milk in Tris-buffered saline with 0.1% v/v Tween 20 (TBS-T). The membrane was incubated with a primary antibody (E-cadherin (1∶2000), Smad1 (1∶1000), Smad5 (1∶1000), Id2 (1∶500), α-SMA (1∶250) and α-tubulin (1∶8000) in TBS-T and either 5% w/v BSA or 5% w/v fat free milk) of interest overnight at 4°C. Following this the blots were washed and incubated with a secondary antibody (goat anti-rabbit HRP conjugate 1∶2000 or anti-mouse HRP conjugate 1∶80000) for 1 h at room temperature. After washing the blots were incubated with western blotting detection reagent (ECL Plus®) for 5 min and the chemiluminescent signal was captured with Amersham Hyperfilm™ ECL (GE healthcare Ltd, Little Chalfont, UK). The membrane was acid stripped and re-probed for other proteins of interest if needed. Scanning densitometry was used to assess the target bands and it was expressed as the ratio of density of target band to a loading control (tubulin).

### Small interfering RNA transfection

The expression of Smad proteins (Smad1, Smad2, Smad3 and Smad5) and Id2 were transiently suppressed by transfecting the cells with validated stealth siRNAs against each target gene. The efficiency of target gene silencing was assessed at the protein level and it was compared against the cells transfected with %GC content matched negative control siRNAs in all the experiments. The cytotoxicity associated with siRNA transfection process was assessed by measuring LDH release at 24 h after the transfection. In all the experiments the cells were transfected when they reached 30–40% confluence. First the transfection mixture was prepared by incubating 2 µl of Lipofectamine 2000 and 100 µl of OPTI-MEM for 5 min at room temperature. Then 40 nmol of siRNA in 100 µl of OPTI-MEM was added to this mixture and it was incubated at room temperature for a further 20 min. Then 800 µl of OPTI-MEM was added to this mixture to make up the total volume to 1 ml and added to the cells. The transfection mixture was removed at the end of 24 h transfection period and the cells were treated with DMEM/F12 (1∶1, v/v) with 5% FCS for 24 h. Before performing growth factor stimulation, the cells were maintained in DMEM/F12 (1∶1, v/v) only without 5% FCS supplement for another 24 h.

### Plasmid transfection

Plasmid vector carrying an insert of Id2-v5 protein coding sequence was used to overexpress Id2 (a kind gift by Dr. Wayne Phillips, Australia). JM109 E-coli (Escherichia Coli) (Cat no JM109, Promega UK Ltd) was used to grow Id2 plasmid and endotoxin free plasmid DNA was prepared by using QIAGEN Endofree™ Plasmid maxi kit (Cat no 12362 QIAGEN UK Ltd) according to the manufacturers instructions. The cells were grown in 35 mm dishes as described under cell culture and the transfection was performed when they reached 50–60% confluence. The transfection reagent complex was prepared by mixing 97 µl of DMEM/F12 (1∶1 v/v) serum free medium with 3 µl of FuGENE® 6 transfection reagent (Cat no 11814443001, Roche Diagnostics Ltd, UK) per 35 mm dish and this was gently mixed and incubated at room temperature for 5 min. 1 µg of plasmid DNA (pcDNA3 Id2-v5 expression vector or pcDNA3.1 empty vector control) per 35 mm dish was added to the transfection reagent complex and this was incubated for a further 30 min at room temperature. 100 µl of transfection complex containing plasmid DNA and 900 µl of fresh medium with 5% FCS was added to the cells and incubated for 24 h. Before growth factor stimulation, the cells were made serum free for 24 h and then treated with TGFβ1 (ng/ml) for 48 h.

### Statistical analysis

All the results are expressed as mean ± standard deviation (SD) of a minimum of 3 experiments. The raw data were blocked using a blocking protocol [33] and analysed by ANOVA with *post hoc* Bonferroni correction using GraphPad PRISM version 3 (GraphPad Software Inc, San Diego, CA, USA) statistical software. P value of <0.05 was considered significant.

## Results

### Id2 was counter-regulated by TGFβ1 and BMP 7

**Figure 1:**
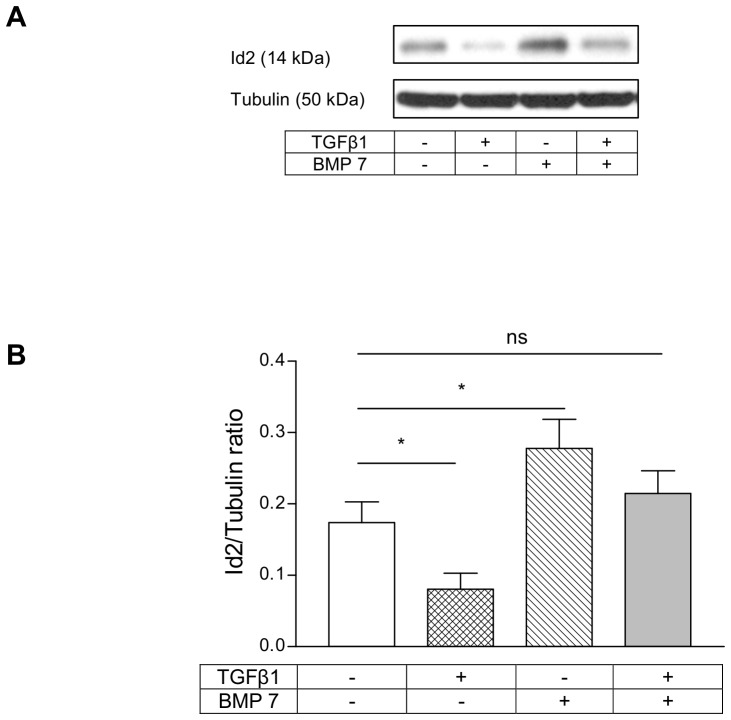
Id2 was counter-regulated by TGFβ1 and BMP 7 in HKC 8 cells. HKC 8 cells were treated with either vehicle (0.1% BSA), TGFβ1 (5 ng/ml), BMP 7 (200 ng/ml) or both for 24 h and the expression of Id2 was determined by immunoblotting. The representative immunoblot shows the expression of Id2. TGFβ1 downregulated Id2 levels on the other hand BMP 7 upregulated Id2 levels and combined treatment of BMP 7 prevented TGFβ1 induced Id2 loss. The data is expressed as mean±SD (n = 3, ns = P>0.05, *<0.05).

The regulation of Id2 expression by TGFβ1 and BMP 7 in HKC 8 cells was studied by treating these cells with TGFβ1 (5 ng/ml) and BMP 7 (200 ng/ml) individually or in combination for 24 h.TGFβ1 downregulated Id2 levels in this cell model (Figure 1). Conversely, BMP 7 upregulated Id2 levels; and when co-treated, BMP 7 prevented TGFβ1 mediated downregulation of Id2 (Figure 1).

### TGFβ1 and BMP 7 regulation of Id2 was Smad mediated

**Figure 2:**
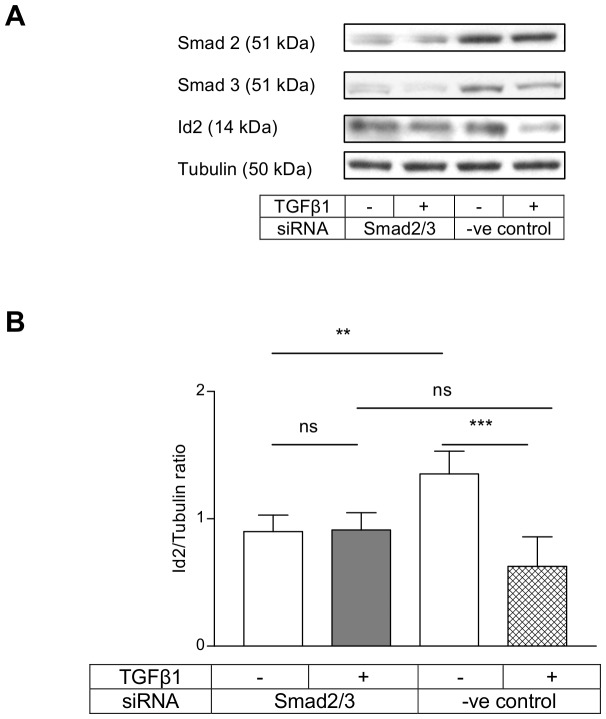
TGFβ1 downregulation of Id2 was prevented by combined Smad2/3 knock-down. HKC 8 cells were transfected with Smad2 and Smad3 siRNAs or negative control siRNA for 24 h. After 24 h serum recovery and 24 h serum free period, the cells were treated with either vehicle (0.1% BSA) or TGFβ1 (5 ng/ml) for a further 24 h. The cells were lysed and the Id2, Smad2 and Smad3 expressions were assessed by immunoblotting. The representative immunoblots show the expression of Id2, Smad2 and Smad3. TGFβ1 downregulation of Id2 was prevented by combined Smad 2/3 knock-down. The data is expressed as mean±SD (n = 6, ns = P>0.05, **<0.01, ***<0.001).

**Figure 3:**
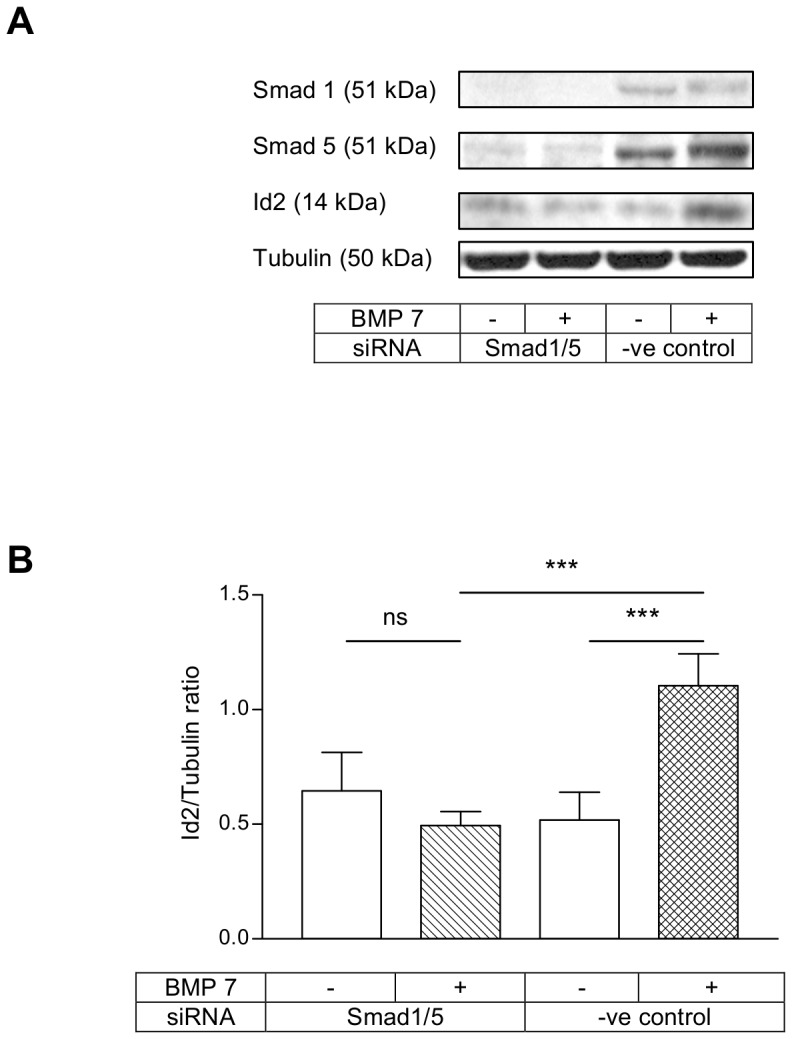
BMP 7 induction of Id2 was prevented by combined Smad1/5 knock-down. HKC 8 cells were transfected with either Smad1 and Smad5 siRNAs or negative control siRNA for 24 h. After 24 h serum recovery and 24 h serum free period, the cells were treated with either vehicle (0.1% BSA) or BMP 7 (200 ng/ml) for a further 18 h. The cells were lysed and Id2, Smad1 and Smad5 expressions were assessed by immunoblotting. The representative immunoblots show the expression of Id2, Smad1 and Smad5. BMP 7 upregulation of Id2 was prevented by combined Smad1/5 knock-down. The data is expressed as mean±SD (n = 5, ns = P>0.05, ***<0.001).

Next we investigated whether the canonical Smad pathway was involved in the regulation of Id2 expression by TGFβ1 and BMP 7 in HKC 8 cells. We used siRNAs to silence the expression of Smad proteins. The siRNAs produced a reproducible inhibition of Smad proteins in all the experiments. TGFβ1 downregulation of Id2 levels was abolished by combined Smad2/3 knock-down (**Figure 2** **A&B**) but not by individual knock-down of either Smad2 or Smad3 (**Figure S1&S2**). Similarly, BMP 7 induction of Id2 was completely abolished by combined Smad1/5 knock-down (**Figure 3** **A&B**) and the individual knock-down of either Smad1 or Smad5 did not prevent this effect (**Figure S3&S4**).

### Id2 inhibition did not contribute to TGFβ1 mediated E-cadherin loss

**Figure 4:**
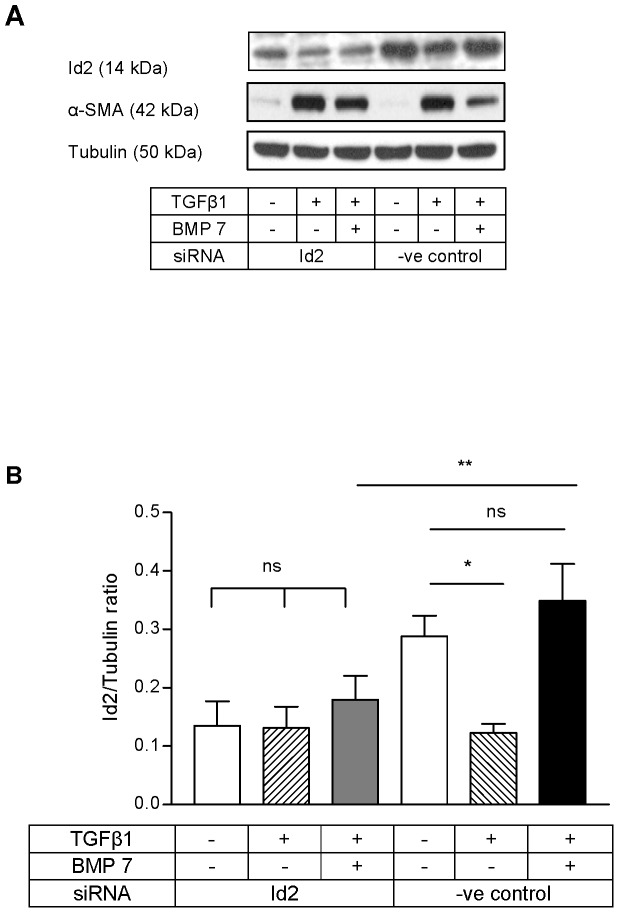
Id2 down-regulation is the crux of α-SMA induction by TGFβ1. HKC 8 cells were transfected either with Id2-v5 expression vector or empty vector for 24 h. After 24 h serum free period they were treated either with vehicle (0.1%BSA) or TGFβ1 (5 ng/ml) for 48 h. E-cadherin, α-SMA and Id2 protein expression were assessed by immunoblotting (A). Id2 over expression did not prevent TGFβ1 suppression of E-cadherin (B). TGFβ1 induction of α-SMA was prevented by Id2 overexpression (C). The data is expressed as mean±SD (n = 4, ns = P>0.05,*<0.05, **<0.01, ***<0.001).

TGFβ1 is well known to induce E-cadherin loss and subsequent EMT in epithelial cell models. The downregulation of E-cadherin by TGFβ1 is primarily mediated by Smad signalling pathway. Similarly the regulation of Id2 by TGFβ1 is also shown to be a Smad mediated event in mouse cell lines. Since TGFβ1 downregulated Id2 by a Smad dependent mechanism, we wanted to investigate whether Id2 is the downstream signal in the Smad2/3 pathway which is activated by TGFβ1 to suppress E-cadherin expression in HKC 8 cells. TGFβ1 treatment of both Id2 overexpressing cells as well as the empty vector transfected cells for 48 h resulted in the downregulation of E-cadherin (**Figure 4** **A,B&D**). The latter suggests that though TGFβ1 downregulated Id2 in HKC 8 cells, it was not required for downregulation of E-cadherin.

### Id2 downregulation is the crux of *de novo* α-SMA induction by TGFβ1

Next we investigated the significance of Id2 downregulation by TGFβ1 on the *de novo* expression of α-SMA; a key Smad mediated event during the EMT of HKC 8 cells. The treatment of HKC 8 cells with TGFβ1 for 48 h resulted in the *de novo* expression of α-SMA in the empty vector transfected cells (**Figure 4** **A,C&D**). However, TGFβ1 induction of α-SMA was prevented by Id2 overexpression and this suggests that Id2 downregulation was required for TGFβ1 induction of α-SMA in this cell model.

### Id2 knock-down did not result in complete EMT with BMP 7 treatment

**Figure 5:**
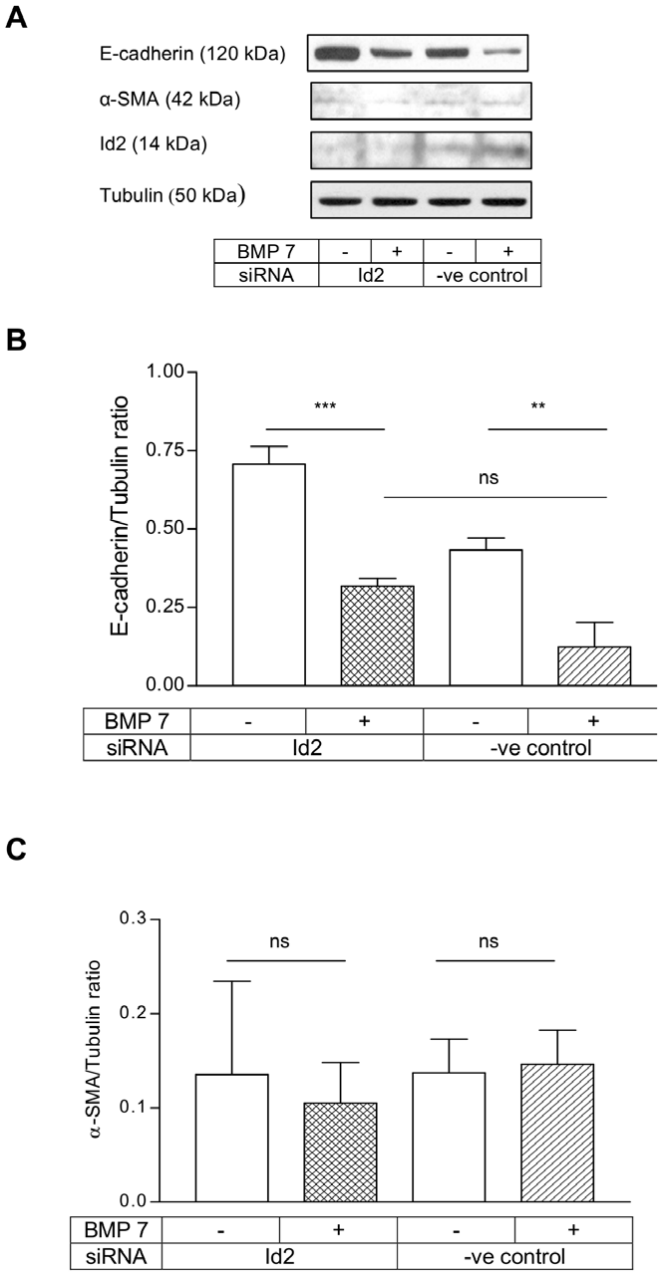
Id2 knock-down did not result in complete EMT with BMP 7. HKC 8 cells were transfected with either Id2 siRNA or %GC content matched negative control siRNA for 24 h. After 24 h serum recovery and 24 h serum free period the cells were treated with either vehicle (0.1% BSA) or BMP 7 (200 ng/ml) for 48 h. The expression of E-cadherin, α-SMA and Id2 were determined by immunoblotting (A). Id2 knock-down did not abolish BMP 7 inhibition of E-cadherin (B). There was no alteration in α-SMA expression with BMP 7 treatment after Id2 knock-down (C). The data (B&C) are expressed as mean±SD (n = 3, ns = P>0.05, **<0.01, ***<0.001).

BMP 7 has been shown to oppose the pro-fibrotic effect of TGFβ1 and one of the mechanisms was inhibition of TGFβ1 mediated EMT. When Id2 was suppressed, BMP 7 itself induced EMT in mouse cell model [22]. We have earlier shown that BMP 7 downregulated E-cadherin but it prevented TGFβ1 mediated α-SMA induction through Smad1/5 signalling. Since BMP 7 regulation of Id2 was also Smad1/5 dependent, we investigated the effect of Id2 knock-down on the expressions of E-cadherin and α-SMA in HKC 8 cells. The Id2 siRNAs produced consistent knock-down in the basal expression of Id2 protein and prevented Id2 upregulation by BMP 7 (**Figure 5** **A**). Id2 knock-down did not have any effect on BMP 7 downregulation of E-cadherin suggesting that Id2 does not mediate BMP 7 inhibition of E-cadherin in HKC 8 cells (**Figure 5** **A&B**). Inhibition of Id2 expression by RNA interference was not sufficient to facilitate BMP 7 induction of α-SMA even in the context of reduced E-cadherin expression (**Figure 5** **A&C**).

### BMP 7 inhibited TGFβ1 mediated α-SMA induction through Id2

**Figure 6:**
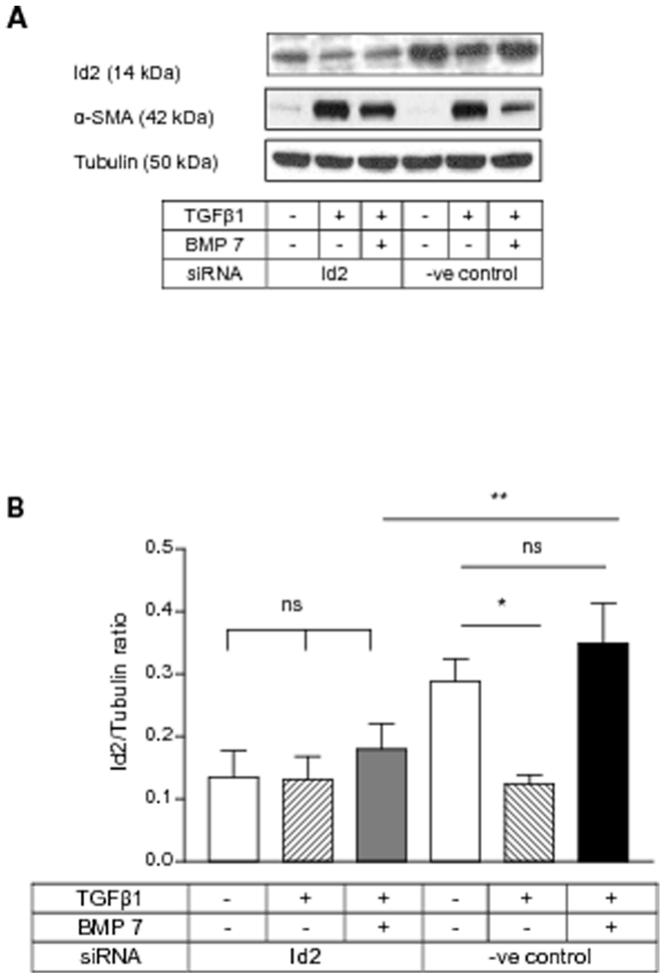
BMP 7 inhibited TGFβ1 mediated α-SMA induction through Id2. HKC 8 cells were transfected with either Id2 siRNA or %GC content matched negative control siRNA for 24 h. After 24 h serum recovery and 24 h serum free period the cells were treated with either vehicle (0.1% BSA), TGFβ1 (5 ng/ml) or TGFβ1 (5 ng/ml) and BMP 7 (200 ng/ml) for 48 h. The expression of Id2 and α-SMA were determined by immunoblotting. The representative immunoblots shows the expression of Id2 and α-SMA (A). Id2 upregulation by BMP 7 was inhibited by siRNA (B). Id2 knock-down prevented BMP 7 inhibition of TGFβ1 mediated α-SMA induction (C). The data is expressed as mean±SD (n = 3, ns = P>0.05, *<0.05, **<0.01, ***<0.001).

Since BMP 7 prevented TGFβ1 mediated Id2 loss and Id2 overexpression prevented *de novo* α-SMA induction by TGFβ1, we investigated whether Id2 regulation by BMP 7 might mediate its anti-fibrotic effect by preventing α-SMA induction and myofibroblastic transition of HKC 8 cells by TGFβ1. Id2 knock-down abolished BMP 7 inhibition of TGFβ1 mediated α-SMA induction (**Figure 6** **A&C**). Id2 upregulation by BMP 7 was inhibited by siRNA (**Figure 6B**).The latter suggests that BMP 7 inhibits *de novo* α-SMA induction by TGFβ1 through restoration of cellular Id2 levels.

## Discussion

The plasticity of the epithelial cells enables them to undergo phenotype switch and participate in development and organogenesis [34]–[36]. Similarly, the phenotypic switch from epithelial cell type to fibroblasts facilitates wound healing process [37]. Although the latter may be beneficial in its limited form, the occurrence of the same in conditions like renal fibrosis is detrimental resulting in excessive matrix accumulation in the interstitium and organ dysfunction. In progressive nephropathies, the interstitium is replaced with matrix secreting fibroblasts in abundance. It was proposed that PTECs are one of the major contributors to this fibroblast population through EMT [11]. Recently Duffield group [7], [17] disputed the role of EMT in renal fibrosis and they proposed pericytes as the major source of fibroblasts. Although the role of EMT of PTECs in the pathogenesis of renal fibrosis is contested, the phenomenon of EMT has been demonstrated unequivocally in human PTEC models *in vitro* [14], [23].

The key features of EMT are the loss of an epithelial marker (E-cadherin) and *de novo* expression of myofibroblast marker (α-SMA). The expressions of these two markers are regulated by bHLH family of transcriptional regulators [22]. Id2, a member of class 4 bHLH proteins has been shown to play a major role in preserving the expression of E-cadherin and a differentiated epithelial phenotype in murine as well as human keratinocytes *in vitro* [21]. Class 1 bHLH factors like E2A gene products (E12 and E47) have been shown to downregulate E-cadherin expression [38], [39] and Id2 inhibited this process. TGFβ1 mediated Id2 inhibition resulted in molar excess of E2A products in NMuMG cells and formation of E2A product dimers leading to E-cadherin loss [21]. In contrast to Id2, Id1 was upregulated by TGFβ1 and this was shown to be responsible for E-cadherin loss, a key step in the initiation of EMT of HKC 8 cells [14]. Id1 when present in abundance formed dimers with HEB, another bHLH protein and inhibited its DNA binding. HEB normally enhances the expression of E-cadherin in these cells and Id1 mediated sequestration of HEB resulted in loss of E-cadherin in the context of TGFβ1 stimulation.

Similar to E-cadherin, α-SMA gene expression has been shown to be regulated by E12, E47and E2-2 [20]. TGFβ1 is shown to induce α-SMA through Smad2/3 signalling, but the downstream pathway regulated by phospho-Smad2/3 in controlling α-SMA expression has not been fully elucidated [23]. Similarly, it is unclear whether TGFβ1 could control α-SMA expression through regulating Id2 in a human epithelial cell type relevant to renal fibrosis. In contrast to TGFβ1, BMP 7 another member of TGF super-family has gained significant interest in the context of fibrosis due to its ability to prevent TGFβ1 mediated fibrosis involving kidney [24], [25], liver [40] and lens capsule [41]. BMP 7 exerts its anti-fibrotic and reparative effects through a number of mechanisms and this includes inhibition of TGFβ1 mediated EMT [24], inhibition of excessive extracellular matrix accumulation [42], enhanced matrix degradation through enhanced MMP 2 and MMP 7 activity [43] as well as inducing proliferation of epithelial cells [44]. We did not find BMP 7 directly inhibiting phosphorylation of TGFβ1 activated Smads in HKC 8 cells (data not shown).

Though BMP 7 was shown to prevent TGFβ1 mediated E-cadherin loss and the subsequent EMT in mouse renal tubule epithelial cells [24], similar results were not consistently reproduced in human models [27]. We and others earlier reported in 2 different human renal PTEC models that BMP 7 did not prevent TGFβ1 mediated E-cadherin loss [28]. Indeed BMP 7 itself downregulated E-cadherin levels in these cell models. However we have shown that BMP 7 inhibited TGFβ1 mediated α-SMA expression and fibronectin production (features of matrix secreting myofibroblasts) in a Smad1/5 dependent mechanism in human PTECs. Again the downstream signalling mechanism responsible for BMP 7 inhibition of α-SMA activation is unknown. Members of BMP family have been shown to regulate Id proteins but the specific role of Id2 in the regulation of human PTEC phenotype is unclear. In particular, whether Id2 may mediate BMP 7 downregulation of E-cadherin and inhibition of α-SMA in PTECs has not been explored.

Hence we investigated the expression of Id2 and its regulation by TGFβ1 and BMP 7 in HKC 8 cells. Id2 was expressed by these cells and was downregulated by TGFβ1. Then we investigated whether this was mediated by Smad signalling. The siRNA mediated silencing of either Smad2 or Smad3 did not abolish the inhibitory effect of TGFβ1 on Id2 expression, but combined Smad2/3 knock-down prevented Id2 loss with TGFβ1 treatment. In contrast to TGFβ1, BMP 7 not only upregulated Id2 but when co-treated with TGFβ1, restored Id2 levels to baseline. The stimulatory effect of BMP 7 on Id2 required both Smad1/5 signalling and the individual knock-down of either Smad1 or Smad5 did not prevent Id2 induction by BMP 7.

Although TGFβ1 repressed Id2 in HKC 8 cells in a similar manner to that seen in NMuMG cells, in HKC 8 cells, unlike NMuMG cells [21], restoration of Id2 levels did not prevent TGFβ1 induced E-cadherin loss. This demonstrates that Id2 is not involved in E-cadherin regulation and initiation of TGFβ1 driven EMT of PTECs. This is not surprising for two reasons, first it is consistent with our previous work where we demonstrated that TGFβ1 mediated E-cadherin repression in HKC 8 cells is Smad3 mediated [23] unlike Id2 regulation which requires both Smad2 and Smad3. Secondly, in PTECs Id1 and not Id2 has been shown to regulate E-cadherin during TGFβ1 mediated EMT [14]. In contrast to E-cadherin expression, Id2 overexpression prevented TGFβ1 mediated α-SMA expression and this is likely mediated by the sequestration of bHLH proteins like E2A products or E2-2 which are involved in the regulation of α-SMA gene transcription.

The work presented here and the previously published work cited above highlights the specificity of Id1 and Id2 in binding to and inhibiting bHLH proteins. In human PTECs, TGFβ1-induced upregulation of Id1 and its subsequent inhibition of bHLH protein DNA binding, selectively regulates E-cadherin while concurrent downregulation of Id2 and consequent increase of bHLH protein DNA binding selectively upregulates TGFβ1-induced α-SMA expression. There is evidence to suggest that Id2 targets E2A proteins, proven regulators of α-SMA [14] and Id1 targets HEB as demonstrated by Li et al [18] This would suggest a pivotal; role for both Id1 and 2 in regulating epithelial phenotype in these cells. Our results with BMP 7 could further support this hypothesis. BMP7 is antagonistic to TGFβ1 and, indeed in this model was shown to upregulate Id2 and prevent TGFβ1 mediated α-SMA expression.

BMP 7 alone had no effect on α-SMA expression both in wild-type PTECs and in the presence of Id2 knockdown, but interestingly it did induce α-SMA in the presence of Id1 knock-down [28].This may be due to the fact that the affinity of interaction between members of Id family and the other bHLH proteins may be variable. The latter, the intracellular localisation and the molar ratio of available cellular Id protein levels in turn could control the transcriptional regulatory activity suggesting a more complex model than proposed above. Langlands K *et al* [45] utilised a yeast 2-hybrid system to analyse the interaction between Id proteins and class I and class II bHLH proteins as well as the ability of Id proteins to disrupt the class I and class II heterodimers. In their model they demonstrated differing affinities of Id proteins for bHLH partners and a consequent difference in their ability to disrupt bHLH dimers.

The inhibition of TGFβ1 mediated α-SMA expression by Id2 overexpression and BMP 7 antagonising α-SMA induction by TGFβ1 through restoring Id2 levels suggests the major involvement of Id2-bHLH protein-E-box axis in the generation of myofibroblastic phenotype of HKC 8 cells. However the specific bHLH factor inhibited by Id proteins in this cell model needs to be established. Similarly these findings need to be confirmed in other PTEC models, primary human PTECs as well as *in vivo* before extrapolating their relevance to the pathogenesis of renal fibrosis in humans given the controversy surrounding the relevance of EMT in the pathogenesis of renal fibrosis.

## Conclusion

The results presented in this work for the first time demonstrate Id2 regulation as the key cellular signalling mechanism responsible for α-SMA induction by TGFβ1 in human PTECs as well as the mechanism downstream of Smad1/5 activated by BMP 7 to inhibit TGFβ1 induction of α-SMA. Although previous reports suggest that loss of E-cadherin is the initiating event and the acquisition of α-SMA occurred as a sequential event during TGFβ1 mediated EMT, we have shown that α-SMA expression and mesenchymal transition could be inhibited even though E-cadherin remained suppressed during TGFβ1 treatment. The latter suggests that E-cadherin loss and α-SMA acquisition are distinct cellular events occurring in a temporal association due to concerted activation of discordant pathways and they could be manipulated individually to prevent the development of pro-fibrotic phenotype.

## Supporting Information

Figure S1 **TGFβ1 downregulation of Id2 was not prevented by Smad2 knock-down.** HKC 8 cells were transfected with Smad2 siRNA or negative control siRNA for 24 h. After 24 h serum recovery and 24 h serum free period, the cells were treated with either vehicle (0.1% BSA) or TGFβ1 (5 ng/ml) for a further 24 h. The cells were lysed and the Id2 and Smad2 expressions were assessed by immunoblotting. The representative immunoblots show the expression of Id2, Smad2. TGFβ1 downregulation of Id2 was not prevented by Smad 2 knock-down. The data is expressed as mean±SD (n = 4, *P<0.05).
(DOC)

Figure S2 **TGFβ1 downregulation of Id2 was not prevented by Smad3 knock-down.** HKC 8 cells were transfected with Smad3 siRNA or negative control siRNA for 24 h. After 24 h serum recovery and 24 h serum free period, the cells were treated with either vehicle (0.1% BSA) or TGFβ1 (5 ng/ml) for a further 24 h. The cells were lysed and the Id2 and Smad3 expressions were assessed by immunoblotting. The representative immunoblots show the expression of Id2, Smad3. TGFβ1 downregulation of Id2 was not prevented by Smad3 knock-down. The data is expressed as mean±SD (n = 7, *P<0.05).
(DOC)

Figure S3 **BMP 7 induction of Id2 was not prevented by Smad1 knock-down.** HKC 8 cells were transfected with either Smad1 siRNAs or negative control siRNA for 24 h. After 24 h serum recovery and 24 h serum free period, the cells were treated with either vehicle (0.1% BSA) or BMP 7 (200 ng/ml) for a further 18 h. The cells were lysed and Id2 and Smad1 expressions were assessed by immunoblotting. The representative immunoblots show the expression of Id2 and Smad1. BMP 7 upregulation of Id2 was not prevented by Smad1 knock-down. The data is expressed as mean±SD (n = 6, *P<0.05, **<0.01).
(DOC)
