# Supplementary figures and images for: Smad Mediated Regulation of Inhibitor of DNA Binding 2 and Its Role in Phenotypic Maintenance of Human Renal Proximal Tubule Epithelial Cells
Source: PLoS One. 2013 Jan 8;8(1):e51842. doi: 10.1371/journal.pone.0051842 (PMC3540025; doi:10.1371/journal.pone.0051842)

**Figure S1**


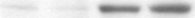


Smad2 (51 kDa)

Id2 (14 kDa)

Tubulin (50 kDa)


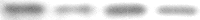


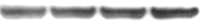


| TGFβ1 | - | + | - | + |
| --- | --- | --- | --- | --- |
| siRNA | Smad2 | | -ve control | |

*

*

| TGFβ1 | - | + | - | + |
| --- | --- | --- | --- | --- |
| siRNA | Smad2 | | -ve control | |

Supplement: Figure S1 — TGFβ1 downregulation of Id2 was not prevented by Smad2 knock-down. HKC 8 cells were transfected with Smad2 siRNA or negative control siRNA for 24 h. After 24 h serum recovery and 24 h serum free period, the cells were treated with either vehicle (0.1% BSA) or TGFβ1 (5 ng/ml) for a further 24 h. The cells were lysed and the Id2 and Smad2 expressions were assessed by immunoblotting. The representative immunoblots show the expression of Id2, Smad2. TGFβ1 downregulation of Id2 was not prevented by Smad 2 knock-down. The data is expressed as mean±SD (n = 4, *P<0.05). (DOC) [file pone.0051842.s001.doc]

**Figure S2**

**
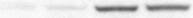

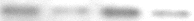

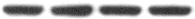
**

Smad3 (51 kDa)

Id2 (14 kDa)

Tubulin (50 kDa)

| TGFβ1 | - | + | - | + |
| --- | --- | --- | --- | --- |
| siRNA | Smad3 | | -ve control | |

*

*

| TGFβ1 | - | + | - | + |
| --- | --- | --- | --- | --- |
| siRNA | Smad3 | | -ve control | |

Supplement: Figure S2 — TGFβ1 downregulation of Id2 was not prevented by Smad3 knock-down. HKC 8 cells were transfected with Smad3 siRNA or negative control siRNA for 24 h. After 24 h serum recovery and 24 h serum free period, the cells were treated with either vehicle (0.1% BSA) or TGFβ1 (5 ng/ml) for a further 24 h. The cells were lysed and the Id2 and Smad3 expressions were assessed by immunoblotting. The representative immunoblots show the expression of Id2, Smad3. TGFβ1 downregulation of Id2 was not prevented by Smad3 knock-down. The data is expressed as mean±SD (n = 7, *P<0.05). (DOC) [file pone.0051842.s002.doc]

**Figure S3**

**
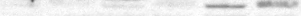
**

Smad1 (51kDa)

Id2 (14kDa)

Tubulin (50kDa)


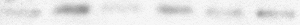


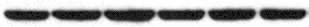


-ve control

Smad1

Smad1

+

+

+

siRNA

-

-

-

BMP 7

*

**

*

-ve control

Smad1

Smad1

+

+

+

siRNA

-

-

-

BMP 7

Supplement: Figure S3 — BMP 7 induction of Id2 was not prevented by Smad1 knock-down. HKC 8 cells were transfected with either Smad1 siRNAs or negative control siRNA for 24 h. After 24 h serum recovery and 24 h serum free period, the cells were treated with either vehicle (0.1% BSA) or BMP 7 (200 ng/ml) for a further 18 h. The cells were lysed and Id2 and Smad1 expressions were assessed by immunoblotting. The representative immunoblots show the expression of Id2 and Smad1. BMP 7 upregulation of Id2 was not prevented by Smad1 knock-down. The data is expressed as mean±SD (n = 6, *P<0.05, **<0.01). (DOC) [file pone.0051842.s003.doc]
